# Aphis Glycines Virus 2, a Novel Insect Virus with a Unique Genome Structure

**DOI:** 10.3390/v8110315

**Published:** 2016-11-19

**Authors:** Sijun Liu, Diveena Vijayendran, Yuting Chen, Bryony C. Bonning

**Affiliations:** Department of Entomology, Iowa State University, Ames, IA 50011, USA; sliu@iastate.edu (S.L.); diveena@genetics.med.harvard.edu (D.V.); yutingc@iastate.edu (Y.C.)

**Keywords:** insect virus, soybean aphid, plant virus, readthrough domain, small RNA virus

## Abstract

The invasive soybean aphid, *Aphis glycines*, is a major pest in soybeans, resulting in substantial economic loss. We analyzed the *A. glycines* transcriptome to identify sequences derived from viruses of *A. glycines*. We identified sequences derived from a novel virus named Aphis glycines virus 2 (ApGlV2). The assembled virus genome sequence was confirmed by reverse transcription polymerase chain reaction (RT-PCR) and Sanger sequencing, conserved domains were characterized, and distribution, and transmission examined. This virus has a positive sense, single-stranded RNA genome of ~4850 nt that encodes three proteins. The RNA-dependent RNA polymerase (RdRp) of ApGlV2 is a permuted RdRp similar to those of some tetraviruses, while the capsid protein is structurally similar to the capsid proteins of plant sobemoviruses. ApGlV2 also encodes a larger minor capsid protein, which is translated by a readthrough mechanism. ApGlV2 appears to be widespread in *A. glycines* populations and to persistently infect aphids with a 100% vertical transmission rate. ApGlV2 is susceptible to the antiviral RNA interference (RNAi) pathway. This virus, with its unique genome structure with both plant- and insect-virus characteristics, is of particular interest from an evolutionary standpoint.

## 1. Introduction

Aphids are major pests of agricultural crops, causing damage to plants by feeding on plant phloem and by the transmission of plant viruses [1,2]. The soybean aphid, *Aphis glycines* Matsumura was introduced into North America in 2000 and has since become a primary pest of soybeans [3]. The estimated losses resulting from reduced soybean yield combined with the cost of management of the soybean aphid in North America are significant [4]. Current management strategies for *A. glycines* populations depend primarily on the use of chemical insecticides. Breeding of soybean cultivars that encode *A. glycines* resistance genes (the “resistance to *A. glycines*” or *Rag* lines) show promise, but aphid biotypes resistant to those cultivars have been identified [5]. Identification of viruses that infect *A. glycines* will allow for investigation of their potential for use in management of *A. glycines*.

Multiple viruses that infect aphids causing serious pathology and disease have been identified. These include viruses with positive sense, single-stranded RNA (+ssRNA) genomes such as the dicistroviruses, e.g., Aphid lethal paralysis virus (ALPV) [6] and Rhopalosiphum padi virus (RhPV) [7]; an Iflavirus, Brevicoryne brassicae virus [8]; and unassigned small RNA viruses such as Rosy apple aphid virus (RAAV) [9] and Acyrthosiphon pisum virus (APV) [6]. DNA viruses (Densoviruses) have also been isolated from aphids [10]. Viral disease in *A. glycines* has not been reported, although sequences of the common aphid viruses ALPV and RhPV were found in the *A. glycines* transcriptome [11].

Next-generation sequencing (NGS) technology has been widely used for the discovery of insect viruses, and is particularly useful for the identification of viruses that replicate in hosts without disease symptoms [12,13]. Many novel insect viruses have been discovered by analyzing NGS data from various species [2]. We analyzed the *A. glycines* transcriptome for assembly and identification of viral genomic sequences. A novel (+) ssRNA insect virus, named Aphis glycines virus 2 (ApGlV2), was discovered. In addition to being a new aphid virus, ApGlV2 has a unique and unusual genomic structure. It is notable that ApGlV2 has a permuted RNA-dependent RNA polymerase (RdRp), similar to those of some tetraviruses and Drosophila A virus (DAV) [14,15,16], while the structure of the coat protein resembles that of plant viruses. We also report on the distribution, transmission, RNA degradation, and biological properties of ApGlV2.

## 2. Materials and Methods

### 2.1. Aphid Rearing

Soybean aphid, *A. glycines* Matsumura populations were collected from soybean fields in Iowa, Michigan, and Ohio, USA. Soybean aphids were reared on soybean plants, *Glycine max* (Williams 82 cultivar) at room temperature (20 °C to 25 °C).

### 2.2. ApGlV2 Genome Assembly

The methods used for assembly of *A. glycines* transcriptome sequence data generated using Illumina sequencing (Illumina, San Diego, CA, USA) have been described [11]. Briefly, total RNA was extracted from *A. glycines* (whole aphids); one RNA sample was prepared with a single round of poly(A) purification (which depletes 50%–75% of RNA without poly(A) tails) and a second sample was prepared with the standard two rounds of poly(A) purification. cDNA was synthesized according to Illumina protocols. The sequencing reads were trimmed to remove adaptor sequences, and reads assembled using the Velvet assembler (European Bioinformatics Institute, Hinxton, Cambridgeshire, UK [7]. The resulting contigs were used for Basic Local Alignment Search Tool (BLAST) analysis against the National Center for Biotechnology Information (NCBI) nr database by using the Blast2GO platform [8]. Contigs with viral genes as top hits were extracted. The contigs with viral hits were then used as subject sequences and the total contigs as query sequences for local BLAST to search for potential additional homologous RNA sequences derived from viruses. The final ApGlV2 genomic sequences were generated manually by reassembling the contigs. The Trinity assembler was also tested for ApGlV2 assembly using default settings [19].

### 2.3. Establishment of Single Soybean Aphid Families

Sequence data suggested that three viruses, ALPV [20], RhPV, and ApGIV2, were present in the laboratory colony of *A. glycines*. In order to isolate *A. glycines* infected only by ApGlV2, we established single-family aphid colonies. These colonies were also used to study the vertical transmission of ApGlV2. To establish RhPV- and ALPV-free single family aphid colonies, individual adult aphids were maintained in petri dishes containing a moist Kimwipe^®^ (Kimberly-Clark Inc., Mississauga, Ontario, Canada) and a *G. max* leaf. The aphids were monitored for production of progeny. The neonates were immediately isolated at birth before contact with the leaf and individually cultured on *G. max* leaves in petri dishes to the adult stage. The isolated aphids were then individually transferred to a *G. max* plant to establish single-family aphid colonies. Aphids from the F2 generation were tested for the presence of ApGlV2, RhPV and ALPV by RT-PCR using primers detailed in Table 1.

To assess whether ApGlV2 was transmitted horizontally via the plant, leaf surfaces were treated with bleach (10%) to eliminate honeydew and aphid contamination. Bleach-treated leaves were washed with water before being used for RNA isolation and RT-PCR for detection of ApGlV2 sequences.

### 2.4. ApGlV2 Virion Purification

To isolate ApGlV2 virions, *A. glycines* infected with only ApGlV2 (1.5 to 3 g) were ground in liquid nitrogen. The homogenate was re-suspended in 20 mL 0.01 M sodium phosphate buffer (137 mM NaCl, 2.7 mM KCl, 10 mM Na_2_HPO_4_, 1.8 mM KH_2_PO_4_, pH 7.0) and centrifuged at 5000 rpm for 5 min in a F21-8X50y rotor using a Sorvall RC-6f centrifuge (Thermo Scientific, Waltham, MA, USA). The supernatant was collected and diluted using 0.3 volumes of chloroform and isoamyl alcohol (24:1), followed by centrifugation at 8000 rpm for 10 min. Virions were precipitated overnight using 2.3% NaCl and 7% polyethylene glycol (PEG; 8000 (*w*/*v*)), followed by centrifugation at 12,000 rpm for 30 min. Virons were resuspended in 2 mL TES buffer (10 mM Tris-HCl, pH 7.5, 2 mM EDTA, 150 mM NaCl), and PEG was removed by centrifugation at 13,000×g for 30 min twice. The virus supernatant was passed through a 20% sucrose cushion by centrifugation at 55,000 rpm for 4 h (Beckman Coulter rotor TLA 120.2, Indianapolis IN, USA). The virion pellet was resuspended in 50 µL TES buffer. All purification and centrifugation steps were conducted at 4 °C.

### 2.5. Characterization of ApGlV2 CP and CP-RTD Proteins

To identify ApGlV2 coat proteins, purified ApGlV2 samples were subjected to 15% sodium dodecyl sulfate polyacrylamide gel electrophoresis (SDS-PAGE) and the gel stained with Coomassie brilliant blue. Individual protein bands were cut from the gel and used for peptide sequencing using in-gel digestion and peptide mass fingerprinting. Digested peptides were analyzed by mass spectrometry (MS) or MS/MS at the Iowa State University Protein Facility (Ames, IA, USA). All MS/MS samples were analyzed using Mascot (version 2.2.0.7, Matrix Science, London, UK), and Sequest HT using Proteome Discoverer 1.4 (Thermo Scientific, Waltham, MA, USA) with the translated soybean aphid transcriptome, or ApGlV2 structural proteins as reference assuming digestion with enzyme trypsin.

### 2.6. Transmission Electron Microscopy

Purified virions (10 µL), were pipetted onto a carbon film grid. The grid was negatively stained with 2% aqueous uranyl-acetate for 30 s. The virus particles were visualized using a JEOL 2100 scanning/transmission electron microscope (JEOL USA, Inc, Peabody, MA, USA) using standard procedures.

### 2.7. RNA and DNA Purification from Aphids

Total RNA from aphids, *G. max* or viral RNA from ApGlV2 virions was isolated using TRIzol^®^ reagent (Invitrogen, Carlsbad, CA, USA) following the manufacturer’s instructions. Aphids (~100 µL) were ground in 1 mL of TRIzol^®^ reagent using a pestle in a microcentrifuge tube. For isolation of viral RNA, 100 µL of purified virus was mixed with 1 mL of TRIzol^®^ reagent in a microcentrifuge tube, briefly vortexed and incubated at 70 °C for 10 min. For extraction of plant RNA, 2 to 3 *G. max* leaves were grouped together for each sample tested. The leaves were homogenized using a mortar and pestle in liquid nitrogen. The homogenate was mixed with 1 mL TRIzol^®^ reagent. The RNA purification protocol followed the manufacturer’s instructions with the modification of overnight RNA precipitation in isopropanol at −80 °C. To purify DNA from *A. glycines*, 0.1 mL of all stages of *A. glycines* were ground in 800 µL of DNAzol^®^ (Invitrogen) as previously described for RNA extraction. RNA and DNA samples were re-suspended in nuclease free water and concentrations determined using a spectrophotometer. Aphid small RNA was isolated from total RNA purified as described above. Libraries of small RNA (sRNA; 17–30 nt) were prepared and sequenced at the Iowa State University DNA Facility using the TruSeq Small RNA Library Preparation Kit (Illumina) and Illumina sequencing. The sRNAs were sequenced for 50 cycles using an Illumina GA II platform.

### 2.8. PCR, RT-PCR, and RACE-PCR

Primers used for detection of viruses and for cDNA sequencing are listed in Table 1. For detection of potential ApGlV2 sequences in aphid genomic DNA [21], total DNA from *A. glycines* was used for PCR with 13 primer combinations that spanned the entire length of the ApGlV2 genome. The PCR was carried out using Choice-Taq™ DNA polymerase (Denville Scientific, South Plainfield, NJ, USA) with 1 cycle of 94 °C for 3 min, 35 cycles of 94 °C for 30 s, 55 °C for 30 s, 72 °C for 3 min and 1 cycle of 72 °C for 10 min. The PCR was repeated with a reduced annealing temperature of 50 °C to amplify products with low melting temperature primers. RT-PCR was used for generation of viral cDNA fragments for either direct sequencing of the purified PCR fragments or for sequencing of the cloned PCR fragments. One-step RT-PCR was used for synthesis of cDNA using the One-Step RT-PCR reaction kit (Qiagen, Hilden, Germany). Viral RNA (100 ng) was added to 10 µL of RT-PCR mix with gene specific primers. The RT-PCR reaction was carried out with one cycle of 50 °C for 30 min, one cycle of 95 °C for 15 min, 30 cycles of 94 °C for 30 s, (lowest Tm for primer −5 °C) for 30 s, 72 °C for 2 min and one cycle of 72 °C for 10 min.

The ends of the virus genome and putative subgenomic RNA were determined using rapid amplification of cDNA ends (RACE) with the SMARTer RACE cDNA amplification kit (Clontech, Mountain View, CA, USA). As ApGlV2 lacks a 3′ end poly (A), the tailing reaction was carried out using poly (A) polymerase (Clontech). The 3′ RACE ready cDNA was generated using RNA with 500 ng of poly (A) tail added and the 5′ RACE ready cDNA was generated using 3 µg of *A. glycines* total RNA, or RNA extracted from purified virions. The Advantage 2 PCR kit (Clontech) was used for the PCR reaction. For RACE, a touchdown PCR reaction was carried out for five cycles of 94 °C for 30 s and 72 °C for 3 min, five cycles of 94 °C for 30 s, 70 °C for 30 s and 72 °C for 3 min followed by 25 cycles of 94 °C for 30 s, 68 °C for 30 s, and 72 °C for 3 min.

All PCR, RT-PCR, and RACE-PCR were performed in a MyCycle Thermal Cycler (BioRad, Hercules, CA, USA). The resulting PCR, RT-PCR, and RACE-PCR products, when necessary, were isolated and purified from 1% agarose gels using the QIAquick gel extraction kit (Qiagen). The purified DNA fragments were either sequenced directly, or ligated into pGEM-T Easy (Promega, Madison, WI, USA). The plasmids were transformed into TOP 10 competent cells. Plasmids were isolated using the Epoch^®^ Biolabs miniprep kit according to the manufacturer’s instructions and sequenced at the Iowa State University DNA Facility.

### 2.9. Bioinformatics Analysis

Local BLAST and pairwise sequence alignments of RNA sequences were performed using the BioEdit Sequence Alignment Editor (version 7.2.5) [22]. ClustalW multiple alignments and phylogenetic analyses of virus genes were performed using MEGA6.06 [23]. The homology modeling program, LOMET (Local Meta-Threading-Server) was used to predict the putative protein structure of RdRp and capsid protein (CP) with default settings [24]. A Perl script was used to map small RNA reads to the ApGlV2 genome. The script was designed to map only reads with perfectly matched bases to the ApGlV2 genome sequence. Positions and orientation in the ApGlV2 genome were recorded for each mapped read. The predicted molecular mass for viral proteins was calculated using ExPASy (expasy.org). The NCBI conserved domain search engine and ExPasy domain search program [25] were used for identification of protein domains.

The 4850-nt genome sequence for ApGlV2 has been deposited in GenBank, National Center for Biotechnology Information, with accession number KR912180.

## 3. Results

### 3.1. Discovery and Confirmation of the ApGlV2 Genome Sequence

To identify viruses of *A. glycines,* we analyzed the *A. glycines* transcriptome [11]. Short contigs of two known aphid dicistroviruses, ALPV and RhPV were identified [20]. In addition to these two aphid dicistroviruses, we identified contigs potentially derived from new viruses by BLASTx analysis. Four contigs of 1004, 1279, 3147, and 3280 nt had hits to the putative RdRp of either DAV (Unclassified) [6], Euprosterna elaeasa virus (EEV; *Permutotetraviridae*; Genus *Alphapermutotetravirus*) [4], or Thosea asigna virus (TAV; *Permutotetraviridae*; Genus *Alphapermutotetravirus*) [15,26]. The RdRp of EEV, TAV, and DAV share similar protein structures, suggesting the contigs might come from the same evolutionarily derived, unknown virus. These contigs were then used as subject sequences for BLASTn to search for additional putative genomic sequences derived from the virus. Three additional viral contigs of 450–2090 nt were identified. We also used Trinity to assemble the ApGlV2 genome, and obtained ApGlV2 sequence that differed slightly at the 5′- and 3′-UTRs. On combining all assembly results, a genome of 4836 nt was obtained.

**Figure 1:**
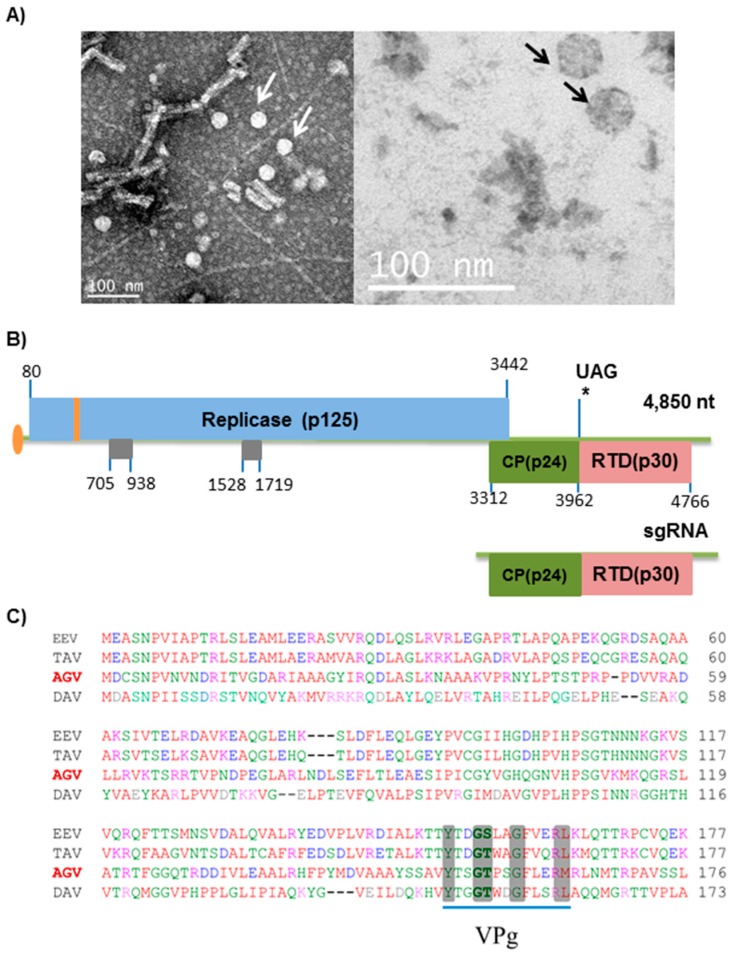
Aphis glycines virus 2 (ApGlV2) morphology and genomic structure. (**A**) Virions of ApGlV2 viewed using a transmission electron microscope. Arrows indicate ApGlV2 icosahedral virions with a diameter of ~30 nm. (**B**) Schematic representation of the ApGlV2 genome. The virus genome likely has a 5′-linked viral protein genome-linked (VPg) with the putative RNA-dependent RNA polymerase (RdRp; 125.3 kDa) encoded by the 5′ open reading frame (ORF). The putative ApGlV2 VPg is encoded at nucleotides 544 to 580 (amino acids 155–167 within the replicase ORF; shown in orange). The capsid protein (CP, 24.3 kDa) is encoded toward the 3′ end along with a putative readthrough domain (RTD, 30.3 kDa). ApGlV2 may encode a subgenomic RNA for the expression of CP and CP-RTD. An additional two potential ORFs (in gray) are located between 705–938 nt (78 aa) and 1528–1719 nt (64 aa). (**C**) The ApGlV2 genome has a conserved VPg domain at the 5′ end. Clustal W sequence alignment of N-terminal sequences of Euprosterna elaeasa virus (EEV), Thosea asigna virus (TAV), Aphis glycines virus 2 (AGV), and Drosophila A virus (DAV). The sequence alignment shows conservation of amino acid sequences (highlighted) for the VPg at the 5′ end of the virus genome.

To confirm the ApGlV2 genome sequence, a colony of *A. glycines* infected with ApGlV2 alone was established. This *A. glycines* colony was tested using RT-PCR with primers specific to ALPV, RhPV, and ApGlV2 to confirm the absence of dicistroviruses and the presence of ApGlV2. ApGlV2 virions were then purified from this *A. glycines* colony and examined using transmission electron microscopy. ApGlV2 virions are icosahedral particles with an estimated diameter of 30 nm (Figure 1A). The putative complete ApGlV2 genome sequence was amplified using RT-PCR with primers designed to span the entire assembled genome of ApGlV2. The genome sequence was confirmed by Sanger sequencing of the amplified RT-PCR products and RACE was conducted to determine the 5′ and 3′ terminal ends of the ApGlV2 genome. The 5′-RACE results indicated a 5′ end 39 nt shorter than the assembled sequence, while the 3′-RACE added an additional 53 nt at the 3′ end of the assembled ApGlV2 genome. However, the 3′ terminal 24 nt of the assembled sequence differed from the 3′-RACE sequence. The final ApGlV2 sequence (with the 3′ sequence determined by RACE) is 4850 nt with 46.6% A + U and 53.4% G + C.

### 3.2. ApGlV2 Genome Organization

ApGlV2 has a single-stranded, positive sense RNA genome; (+)ssRNA. The viral genomic RNA has two predicted open reading frames (ORFs) (Figure 1B). ORF1 (P125) encodes a 1121-amino acid (aa) protein, containing the RdRp domain, predicted to be a replicase. The overlapping ORF2 encodes 217 aa and is predicted to encode the capsid protein (CP, P24). In addition to the two major functional ORFs, an additional 244 aa ORF3 (P30) of unknown function downstream of CP is also predicted. Although ORF3 has an in-frame AUG, it is likely that it is expressed via stop codon readthrough due to the polyproline linker sequence upstream of the first AUG, which is typical for the readthrough domain (RTD) of luteovirid CP-RTD [27]. The stop codon of the CP is UAG, an amber stop codon commonly found in readthrough proteins. Hence the 244 aa ORF3 could be translated by readthrough of the CP and form a 485 aa putative CP-RTD protein (55 kDa) (Figure 1B). The CP stop context conforms to the “Type I” classical readthrough motif, namely UAG-CAR-YYA (R = purine, Y = pyrimidine) (cf. many plant viruses, [28], but also Providence virus, which has UAG-CAA-CUA—the same as ApGlV2). The CP and CP-RTD are likely to be translated from a subgenomic RNA based on the genomic structure, with ORF2 and ORF3 in a +1 frame relative to ORF1. This is the first insect RNA virus described that has a CP-RTD structure similar to that of plant luteovirids. In addition to the three major ORFs shown in Figure 1B, there are two potential ORFs located between 705–938 nt (78 aa) and 1528–1719 nt (64 aa). It is unknown whether these short ORFs are translated.

ApGlV2 appears to lack a poly(A) tail. The near full-length ApGlV2 genome sequence was assembled from NGS reads derived only from partially purified mRNA samples [11], and ApGlV2 sequence was not PCR amplified when reverse transcription was carried out using an oligo d(T) primer. The same region was successfully amplified, however, when a virus-specific RT primer was used (data not shown).

Viruses with RdRp homologous to that of ApGlV2 encode viral protein genome-linked (VPg) at the N-terminus of the RdRp protein. Sequence comparison of TAV, EEV DAV, and ApGlV2 shows that the RdRp of ApGlV2 includes residues consistent with conserved VPg sequences (Figure 1C).

### 3.3. Analysis of Putative Subgenomic RNA

The genome structure of ApGlV2 suggests that CP and CP-RTD are likely translated from a subgenomic RNA (sgRNA). ApGlV2 genomic RNA was unstable. Under conditions in which we could purify intact Aphid lethal paralysis virus RNA (10 kb), ApGlV2 RNA was degraded (Supplementary Materials Figure S1). Hence it was not possible to detect sgRNA by conventional methods. To detect the potential sgRNA, a primer that anneals close to the CP start codon (ApGlV2-sgRNA 5′ RACE, Table 1) was used for 5′-RACE to amplify the 5′-UTR of the sgRNA. The 5′-RACE resulted in various lengths of the 5′-UTR of the putative sgRNA, which were 23, 64, 78, and 111 nt upstream of the CP start codon.

### 3.4. ApGlV2 Encodes a Permuted RdRp

**Figure 2:**
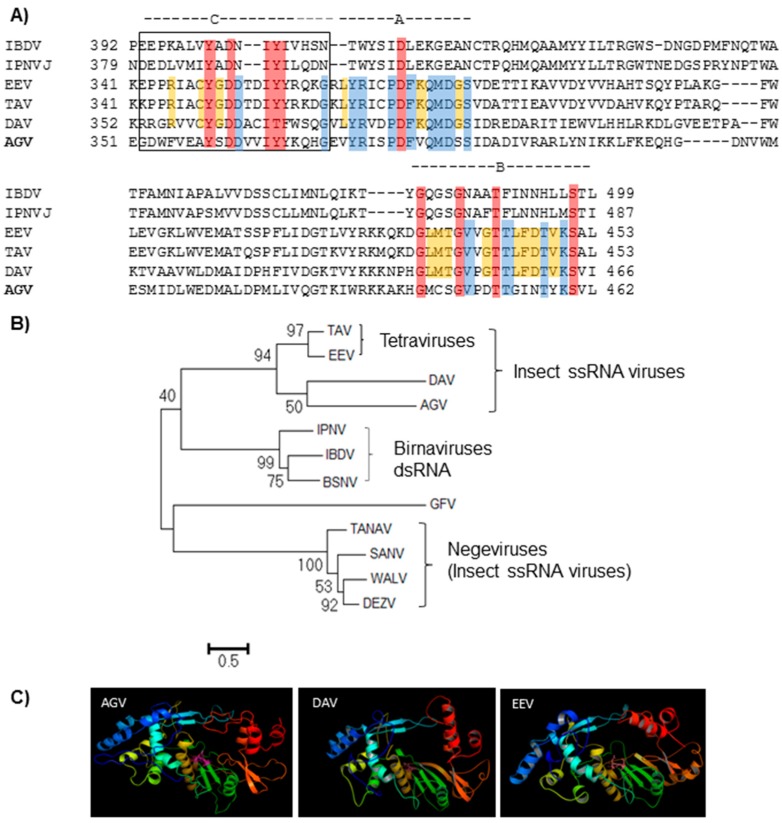
ApGlV2 has a non-canonical (C-A-B) RdRp, similar to those of some other insect viruses. (**A**) The conservation of the amino acid residues between ApGlV2, the tetraviruses; Thosea asigna virus (TAV) and Euprosterna elaeasa virus (EEV); Drosophila A virus (DAV) and birnaviruses; Infectious pancreatic necrosis virus (IPNV) and Infectious bursal disease virus (IBDV) are highlighted. Yellow highlights show conservation between EEV, TAV, and DAV but not ApGlV2 while blue highlights show conservation of EEV, TAV, and DAV with ApGlV2. Red highlights show conservation of residues between all viruses. All highlighted amino acids are key RdRp motifs. (**B**) Phylogenetic tree of ApGlV2 RdRp with closely related viruses and other viruses with permuted RdRp based on BLAST results of amino acid identity. Phylogenetic trees were constructed using MEGA 6.06 with the maximum likelihood method. Thosea asigna virus (TAV, accession AAQ14329.1) and EEV (AF461742_1) are closely related to but phylogenetically distinct from DAV (YP003038595.1) and ApGlV2 (AGV). The RdRps of these insect viruses are clustered with Birnaviruses, e.g., Infectious pancreatic necrosis virus (IPNV; AAV48847.1), Infectious bursal disease virus (IBDV; ACS44343.1), and Blotched snakehead virus (BSNV; YP052864). In contrast, the RdRps of negeviruses with permuted RdRps, Dezidougou virus (DEZV, AFI24669.1), Santana virus (SANV, AFI24675.1), Wallerfield virus (WALV, AIS40860.1), and Tanay virus (TANNV, YP_009028558.1) are close to a plant virus, Grapevine fleck virus (GFV, CAC84400.1), forming a distinct branch and indicating that the RdRps of negeviruses are distinct from that of ApGlV2; (**C**) The predicted RdRp structures for ApGlV2 (AGV), DAV, and EEV are similar. Homology modeling of tertiary protein structures of RdRp from ApGlV2, Drosophila A virus (DAV), and Euprosterna elaeasa virus (EEV). Images were generated using the LOMETS server.

BLAST analysis of ApGlV2 RdRp resulted in hits to TAV, EEV, and DAV, suggesting that ApGlV2 may be a new insect virus with a non-canonical permuted RdRp Sequence comparison between ApGlV2 RdRp and the permuted RdRps of TAV, EEV, and DAV showed that the ApGlV2 RdRp has a non-canonical C-A-B-D arrangement (Figure 2A). Residues of the motifs are conserved among the RdRps of ssRNA insect viruses with permuted RdRps. However, the ApGlV2 motifs are more divergent than those of the other three insect virus permuted RdRps. 

Phylogenetic analysis of the permuted RdRps of insect viruses, birnaviruses, and a plant virus shows three distinct lineages (Figure 2B) with ApGlV2, DAV, and the tetraviruses closely related to the birnaviruses, suggesting that the permuted RdRps of these viruses evolved from a common ancestor (Figure 2B). In contrast, the RdRps of the negeviruses are more closely related to a plant virus, Grapevine fleck virus (GFV). Detailed analysis of the ApGlV2 RdRp showed that although the full-length RdRp showed homology, only the N-terminal half of the ApGlV2 RdRp has significant amino acid similarity to that of EEV, TAV and DAV (query coverage 56%–59% with 32%–33% amino acid sequence identities), i.e., the sequence homology of the viral RdRp is not high at the amino acid level. To determine whether the four RdRps form similar tertiary structures, protein folding of the RdRps was predicted by homology modeling using the LOMETS server. The structure prediction results demonstrate that the RdRp of ApGlV2 has a similar tertiary structure to those of the tetraviruses and DAV (Figure 2C), despite the low sequence similarity among the permuted RdRps of these viruses.

### 3.5. Analysis of ApGlV2 CP and Putative RTD

**Figure 3:**
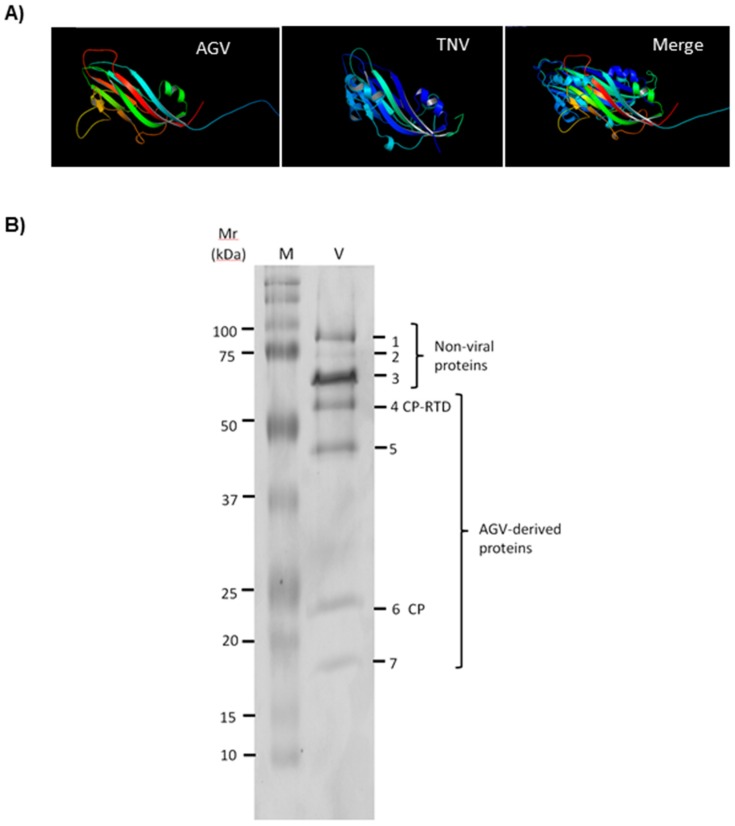
ApGlV2 capsid proteins. (**A**) The predicted structures of ApGlV2 (AGV) and tobacco necrosis virus (TNV) capsid proteins are similar. Homology modeling of tertiary protein structures of viral capsid protein for ApGlV2 and TNV (PBD:1C8N). Merge shows superimposed images of the two predicted CP structures with signature jelly-roll-like symmetric antiparallel β sheets. The images were generated using the LOMETS server. (**B**) Identification of ApGlV2 structural proteins. Purified virions (V; ~5 µg) were subjected to sodium dodecyl sulfate polyacrylamide gel electrophoresis (SDS-PAGE) (15% gel) and bands cut from the Coomassie brilliant blue-stained gel. The seven bands (1–7) were isolated for peptide sequencing. M, molecular mass markers.

The 217 aa ApGlV2 P24 gene is a predicted capsid protein. Remarkably, there were no significant BLAST hits of the CP amino acid sequence to any insect or animal virus. All hits to ApGlV2 CP were from plant viruses, with the top hit (31% amino acid identity and 79% sequence coverage) from a newly identified viral sequence isolated from bat fecal material, the so-called Bat sobemovirus (BSV; NCBI Accession No: AGN73380.1 [29]). Most of the top BLAST hits were from either sobemoviruses or viruses within the *Tombusviridae* (data not shown), suggesting that the ApGlV2 capsid protein is evolutionarily derived from a plant virus. DAV is also an insect virus encoding a plant virus-like CP, and also encodes an RdRp similar to that of ApGlV2 [6]. However, the sequence identity between the CP of ApGlV2 and DAV is only 25%, with sequence coverage of 46%. Homology modeling to determine the tertiary structure of ApGlV2 CP suggests that ApGlV2 CP folds similar to the CP of tobacco necrosis virus (TNV; *Tombusviridae* Figure 3A). The sequence similarity and homology modeling results indicate that ApGlV2 is structurally similar to certain plant viruses (Sobemoviruses and *Tombusviridae*).

A remarkable feature of ApGlV2 is the apparent production of a CP-RTD protein by translational readthrough of the CP stop codon. The 267 aa RTD has a polyproline track at its N-terminus, which may function as a hinge to expose RTD on the virion surface. This type of CP-RTD was previously observed in luteovirids. The luteovirid RTD plays an important role in virus transmission by the aphid vector [30,31] and also functions in virus movement and luteovirid tissue specificity (phloem restriction) in the plant [27,32].

To detect the predicted CP and CP-RTD proteins, ApGlV2 particles were purified in a sucrose cushion, denatured and separated by sodium dodecyl sulfate polyacrylamide gel electrophoresis (SDS-PAGE). Seven protein bands on the gel (Figure 3B) were isolated for peptide sequencing to establish whether the seven protein bands were derived from ApGlV2. Peptide sequences from bands 4–7 matched the ApGlV2 CP sequence, indicating that these four proteins were derived from ApGlV2 CP (Supplementary Materials Figure S2). Sequences obtained from proteins with higher molecular mass (proteins 1–3) showed no similarity to virus sequences on BLAST analysis: Peptide sequences from protein band 3 were derived from *Buchnera aphidicola* 60 kDa chaperonin, while sequences from protein bands 1 and 2 had no significant hits. Peptide sequences obtained for band 4 (~55 kDa) were derived from CP, indicating that band 4 was CP-RTD (predicted Mr 55.5 kDa), while band 6 of ~24 kDa was derived from CP. Unfortunately, no peptides derived from the RTD domain were detected. Protein band 5 was larger than CP but smaller than the predicted CP-RTD and band 7 was smaller than the predicted CP, suggesting these two proteins were truncated forms of CP-RTD and CP. These peptide sequencing results combined with the molecular mass of virus proteins containing CP-derived amino acid sequences are consistent with the predictions that ApGlV2 encodes CP and CP-RTD.

Comparison of the RTD protein sequence with the sequences of other proteins did not result in any significant hits on BLASTp analysis. In addition, no conserved protein domains, family, functional sites, or motifs were identified.

### 3.6. Distribution and Transmission of ApGlV2

ApGlV2 was originally isolated from a laboratory colony of *A. glycines* that had been established with aphids collected from the field in Iowa, USA. The presence of ApGlV2 in field populations of *A. glycines* from Michigan and Ohio, USA was confirmed by RT-PCR amplification of the RdRp region (929 bp product) of ApGlV2 (Supplementary Materials Figure S3).

To determine the mode of transmission of ApGlV2 among individual aphids, we first assessed the prevalence of ApGlV2 within the Iowa-derived laboratory *A. glycines* population by RT-PCR. Ten aphids were randomly isolated and used for RNA isolation. All 10 aphids were positive for ApGlV2. We then assessed the vertical transmission of ApGlV2. Sixty-three individual aphids isolated from 15 adults were tested by RT-PCR for the presence of ApGlV2. All aphids tested positive, suggesting that the virus was 100% vertically transmitted from adults to the newborn nymphs. These results suggested that all individual aphids were infected by ApGlV2. Because all tested aphids were ApGlV2-positive, whether ApGlV2 could also infect aphids by horizontal transmission could not be addressed. It is possible that ApGlV2 can be transmitted via the host plant, similar to other aphid viruses: RhPV, RAAV, Dendrolimus punctatus DNV, and Myzus persicae DNV [33,34]. RT-PCR on RNA isolated from the *G. max* leaves that were infested with aphids was conducted to test whether ApGlV2 is transmitted via the plant. No ApGlV2 RNA was detected by RT-PCR from these leaves, indicating that ApGlV2 was not introduced into the host plants (data not shown).

### 3.7. ApGlV2 Is Not Detected in the Genome of *A. glycines*

Insertion of sequences of persistent insect RNA viruses into the host genome has been reported for Flock house virus (FHV, Nodaviridae) and Israeli acute paralysis virus of bees (IAPV, *Dicistroviridae*) [21,35]. Detection of ApGlV2 in all *A. glycines* samples from multiple locations and the 100% vertical transmission rate prompted testing for the presence of ApGlV2 sequences in the genome of *A. glycines*. To eliminate the possibility that ApGlV2 genomic RNA was inserted into the *A. glycines* genome, we designed combinations of primer pairs spanning the entire genome of the virus and the primers were used in PCR with total DNA isolated from *A. glycines*. None of the sequenced PCR products were derived from the ApGlV2 genome sequence (Supplementary Materials Figure S4). We then mapped NGS DNA reads (100 bp) derived from the same Iowa soybean aphid population from which ApGlV2 was isolated [36] to the ApGlV2 genome. No genomic DNA reads mapped to ApGlV2 genomic RNA. The PCR and genomic mapping results indicate that ApGlV2 sequences were not present in the genome of *A. glycines*.

### 3.8. ApGlV2 Is Targeted by the RNAi Pathway

**Figure 4:**
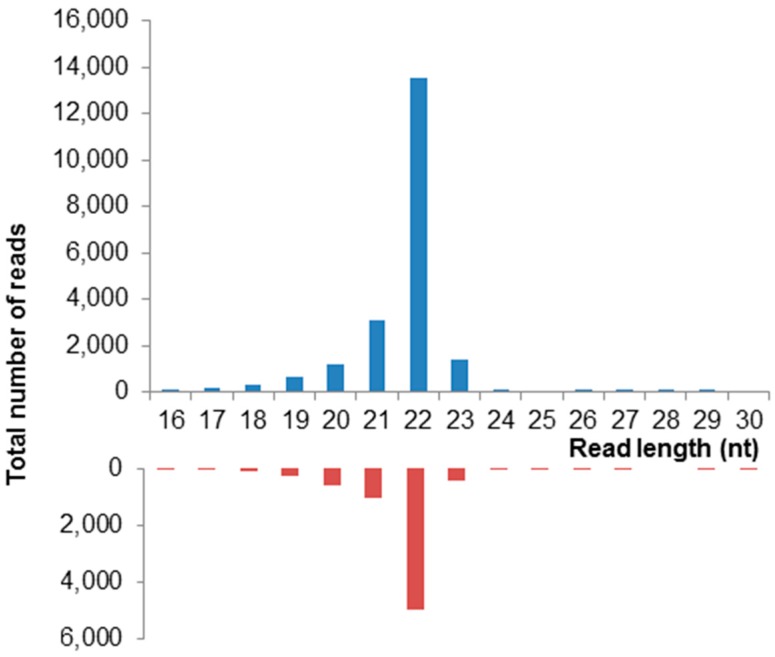
Virus-derived small interfering RNA (vsRNA) sequences derived from the sense (blue) and anti-sense (red) genome of ApGlV2. Reads were extracted from a small RNA sequencing dataset of *A. glycines* (Iowa) infected with ApGlV2 and an ALPV-like virus. The majority of the ApGlV2 vsRNAs are 22 nt, characteristic of double-stranded RNA processing by Dicer2.

The RNA interference (RNAi) pathway plays a critical role in antiviral immune defense for many insect viruses 37]. To determine whether degradation of ApGlV2 is regulated by the RNAi pathway, we sequenced the sRNA of the Iowa *A. glycines* colony infected by ApGlV2 and an ALPV-like virus using the Illumina platform. The sRNA reads (16–30 nt) were mapped to the ApGlV2 genome to detect sRNA derived from ApGlV2. Around 0.4% of the reads (from a total of 20.6 million reads) mapped to ApGlV2 genomic RNA. A typical viral small RNA (vsRNA) distribution pattern with a peak at 22 nt derived from both strands of the dsRNA replication intermediate was observed (Figure 4).

## 4. Discussion

The novel insect virus, Aphis glycines virus 2 (ApGlV2), has a unique genome structure that encodes an RdRp similar to that of insect viruses and a capsid protein resembling those of luteovirids. ApGlV2 infected all individuals in all soybean aphid populations examined and is vertically transmitted. ApGlV2 replication in *A. glycines* was confirmed by sRNA sequencing with virus-derived sRNA identified from both RNA strands, indicative of RNAi-mediated degradation of the viral dsRNA produced during replication. The impact of ApGlV2 infection on the soybean aphid could not be assessed due to the lack of an ApGlV2-free soybean aphid colony.

Viral RdRps are the most conserved viral proteins and share several conserved motifs in a specific order [38,39,40]. The arrangement of the conserved structures has a “right hand-like” shape with three conserved sub-domains, named the finger, palm, and thumb [41,42]. The palm domain consists of four of eight conserved motifs that have a canonical order described as A-B-C-D and found in most viral RdRp [39]. However, a non-canonical organization of the RdRp motifs was observed in seven ssRNA insect viruses: The tetraviruses TAV, EEV, and the unclassified virus DAV [14,15,16]; negeviruses isolated from mosquitoes; DEZV, SANV [43], WALV [44], and Tanay virus, TANAV [45]; three dsRNA viruses (BSNV, IPNV, and IBDV) and a plant alpha-like virus (Grapevine fleck virus, GFV) [15,16,46,47]. More recently, the sequences of five new insect viruses with permuted RdRp have been submitted to NCBI, specifically Newfield virus (NfV) (partial sequence: KP714979.1) [48], Niehaus virus (NhV) (partial sequence; KX580892), Egaro virus (EgV) (NC_030845) Daeseongdong virus (DaV2) (NC_028489) [49], and Defiwi virus (DfwV) (partial sequence; KX580893). The motif C of the RdRp in these viruses is permuted upstream of the motif A to form a non-canonical C-A-B-D arrangement.

ApGlV2 represents a new type of insect virus that also has a permuted RdRp. A permuted RdRp was first found in the insect tetraviruses (EEV and TAV), with the RdRp closely related to those of birnaviruses [15]. On the basis of this, the tetraviruses were reclassified into a new family (*Permutotetraviridae*) and a new genus (*Alphapermutotetravirus*) [14,26]. A permuted RdRp was subsequently found in DAV and Drosophila melanogaster tetravirus SW-2009a (DTRV) [50], which is actually an isolate of DAV [16]. The permuted RdRps of Negevirus, which are homologous to RdRp-2 [45], differ from those of TEV, EAV, DAV, and ApGlV2. Sequence analysis indicated that Negevirus RdRps are closely related to those of a plant virus (Citrus leprosis virus C; CiLV-C) and other plant viruses whose RdRps contain RDR-2 domains (e.g., Hibiscus green spot virus, an unclassified ssRNA virus of Higrevirus and Grapevine fleck virus) [43,45].

In addition to negeviruses, and permutotetraviruses, the recent discovery of novel insect viruses suggests that permuted RdRps have evolved in divergent insect viruses. Sequence comparison suggests that the RdRp of DAV, ApGlV2 and other unassigned viruses [16,48,49] (Liu unpublished results) show some similarities to permutotetraviruses, but not to negeviruses. However, the CP of these viruses are diverse (data not shown).

If the CP and CP-RTD structural protein sequences of ApGlV2 are encoded by the genomic RNA (rather than the sgRNA), the 5′RACE from the CP start codon region would be expected to have gone further than 111 nt. The detection of differing lengths of 5′ UTR is not uncommon for 5′RACE reactions. It is also unlikely that the reverse transcriptase fell off the RNA template during RACE due to secondary structures in the RNA as the entire genomic sequence was confirmed by RT-PCR. Although our results support the hypothesis that the structural proteins of ApGlV2 are encoded by an sgRNA, this remains to be confirmed.

The organization of the structural proteins of ApGlV2 resembles that of luteovirids. Luteovirids are exclusively transmitted by aphids in a persistent and circulative manner but do not replicate in aphids. Low sequence identity of ApGlV2 CP to those of sobemoviruses and other plant viruses suggests that these viruses only share similar virion structures. In addition, except for the polyproline sequences at the N-terminus, the ApGlV2 RTD has no sequence homology to the RTD of luteovirids, an indication that the function for ApGlV2 RTD may differ from that of luteovirids.

Viruses encoding a CP-RTD as a large minor virus coat protein have been found in several RNA virus groups such as luteovirids, benyviruses, and alloleviviruses [51,52,53,54]. One common feature of the CP-RTD is that they all have proline-rich regions at the N-terminus of the RTD. These regions act as relatively rigid spacers to separate the RTD from the CP. In the plant viruses (luteovirids and benyviruses), the RTD is involved in virus transmission by a vector. For instance, the RTD of luteovirids facilitates (but is not essential for) virus entry and circulation in the aphid vector by interaction with the aphid gut and salivary glands [55]. For Beet necrotic yellow vein virus (BNYVV), the type member of benyviruses, the RNA2 encodes a p54 RTD containing a KTER motif that is essential for transmission of the virus by its vector *Polymyxa betae* (reviewed by [56]. Like other viral CP-RTD, the RTD of ApGlV2 is likely exposed on the virion surface.

Interestingly, an aphid glycoside hydrolase, a predicted lactase-phlorizin hydrolase-like protein (LPH; [57], co-purified with ApGlV2 when methods frequently used in isolation of ALPV from the pea aphid *A. pisum* [20] was used for isolation of ApGlV2 (data not shown). No LPH accumulation was observed in preparation of ALPV from *A. pisum*, suggesting that the buffer system used for purification did not result in precipitation of LPH. The biological relevance of this association of ApGlV2 with LPH if any is unclear. Because all *A. glycines* tested were infected with ApGlV2, it was not possible to establish an ApGlV2-free colony to verify whether a mock preparation resulted in LPH precipitation.

While the *A. pisum* genome encodes all major components of the RNAi pathway [58], the role of RNAi in antiviral defense in aphids has not previously been demonstrated. The detection of ApGlV2-derived vsRNA also confirms that ApGlV2 actively replicates in *A. glycines* with a genome that is susceptible to the host antiviral immune response. The sRNA were sequenced from a colony known to be infected by both ApGlV2 and an ALPV-like virus [20]. Dicistrovirus RNAi suppressor proteins are known to inhibit the RNAi pathway. For example, the Drosophila C virus (DCV) encodes DCV-1A that binds to and inhibits the processing of dsRNA by Dicer (Dcr2) while Cricket paralysis virus (CrPV) codes for CrPV-1A which binds to Argonaute (Ago2) in RISC to inhibit the cleavage of the target RNA [59,60]. It is not known whether the presence of ALPV in the Iowa colony had any impact on RNAi-mediated degradation of ApGlV2, or whether ApGlV2 also encodes a suppressor of silencing.

ApGlV2 RNA extracted from purified ApGlV2 virions ran on gels as a smear, while a distinct RNA band was observed for ALPV RNA (Supplementary Materials Figure S1). A similar observation was reported for DAV with DAV RNA degraded rapidly during extraction and electrophoresis [16]. RNA extracted from some tetraviruses is unstable outside of the virion [61].

The current version of the ApGlV2 genome sequence is based on confirmation of the assembled ApGlV2 sequences by RACE. Although the 3′-UTR of ApGlV2 is likely correct, the 5′-end sequence remains uncertain. The assembled ApGlV2 sequence contained an additional 39 nt at the 5′-end. Although repeated 5′-RACE experiments did not confirm this 39 nt, assembly of ApGlV2 from various sequencing sources showed the 39 nt in the 5′-UTR (data no shown). In addition, 5′-RACE of sgRNA showed variable results, suggesting 5′-RACE is not reliable for confirming the 5′-end sequences of ApGlV2. Similar 5′-RACE resulting in shorter 5′-UTR sequences than those of the assembled viral genomes have been reported previously [20,62,63], demonstrating challenges for use of this method for determining viral 5′-UTR sequences [64]. The failure of 5′-RACE could result from RNA secondary structures [63] or because of genome protection by VPg. The rapid degradation of ApGlV2 genomic RNA (Supplementary Materials Figure S1) may also contribute to a lack of 5′-RACE efficiency.

In conclusion, ApGlV2 represents a new virus type with a unique genomic structure, which encodes a permuted RdRp similar to several insect viruses, and structural proteins similar to plant viruses. ApGlV2 RNA is subject to degradation by the RNAi pathway. This study highlights the potential for the use of next-generation sequence data for identification of virus-derived sequences [13] and some of the challenges associated with confirmation of sequences identified in silico [64,65].

## Figures and Tables

**Table 1:** Primers used for reverse transcription PCR (RT-PCR) and rapid amplification of cDNA ends (RACE) PCR for ApGlV2, ALPV, and RhPV in *A. glycines* and for PCR to test for the presence of ApGlV2 sequences in the aphid genome. F denotes a forward primer while R denotes a reverse primer. N/A, not applicable.

Primer Name	Sequence (5′–3′)	ApGlV2 Genome Position
Tet-F	AGTGGCTGCGCATGCTCGTT	1695–1714
Tet-R	ACGCGCCTCTCCGTTGAACT	2604–2623
ApGlV2 3′-F	CAGTACAGCAATACGGCTCATT	4284–4305
ApGlV2 3′-R	AAGGGCCTTATTACTTTTCACACTCTCTC	4474–4502
ApGlV2 5′-F	GCAGGACCTTGCCTCGCTCAAA	160–181
ApGlV2-RdRp-F	AGTGGCTGCGCATGCTCGTT	1695–2014
ApGlV2-RdRp-R	ACGCGCCTCTCCGTTGAACT	2603–2623
ApGlV2 R/C-F	CACGCGCGGAATCTTTGCAG	2976–2995
ApGlV2 R/C-R	TCGGTCTTGGCGGCGTCATA	3825–3845
ApGlV2-CP-F	AGCAGAGCTCAACACGACGAACCAAG	3315–3329
ApGlV2-CP-R	AGCAAGCTTCTAAGCTCTCGTG	3953–3965
ApGlV2 C/T-F	TGTGACTCCGACACCGTCGAA	3885–3905
ApGlV2 C/T-R	GCACCGGGAGAAATCCCAGAGT	4424–4445
ApGlV2 3′ RACE	TCTCCCGGTGCCTCGTCTCACCACAGG	4435–4461
ApGlV2-sgRNA 5′ RACE	CCCGAGAGTTTTATTTATGCTGGTGGACGATATGGGCAGAGACA	3748–3791
ApGlV2 5′ RACE	AAGTGCCGTAGCGCTGCCTCGAGCAC	476–501
ALPV-F	TGAACTTCGTGCAACGAACACTGTT	N/A
ALPV-R	TCCGCCTGCGTTAGGAAGAAGA	N/A
RhPV-F	AATCTGGCGTTGACGCGCTC	N/A
RhPV-R	TCCCCCATCATCAACATAGATGCGT	N/A
qRT-PCR ApGlV2-F	TCCCCGCCACGTGAAGTGAA	2624–2643
qRT-PCR ApGlV2-R	GCTACTGCGTGCGTGGTGAA	2822–2841

## Supplementary Materials

The following are available online at www.mdpi.com/1999-4915/8/11/315/s1, Figure S1: Gel electrophoresis (1% native agarose) of 2 µg RNA extracted from purified ApGlV2 and ALPV-AP virions. While the ALPV-AP RNA runs at the expected size of ~10 kb, the ApGlV2 RNA runs as a smear indicative of RNA degradation. Samples visualized with ethidium bromide staining; Figure S2: Peptide sequences derived from ApGlV2 structural proteins. Peptide numbers correspond to protein bands in Figure 3B; Colors represent sequences of individual peptide fragments. Proteins were identified from these peptide sequences with high confidence based on Mascot search A2 values; Figure S3: Detection of ApGlV2 RdRp sequence from field collected *A. glycines*. The 929 bp RT-PCR product was detected in field collected *A. glycines* from Iowa, Ohio and Michigan, USA. The positive control and *A. glycines* samples amplified the expected PCR products. No amplification was detected in the no template control sample (NTC); Figure S4: ApGlV2 sequences were not detected in the *A. glycines* genome. Primers spanning the entire ApGlV2 genome were used to test for the presence of ApGlV2 sequence in the *A. glycines* genome. A representative agarose gel with amplified PCR products is shown. Labels indicate region of ApGlV2 genome spanned by the PCR primers. CP, capsid protein; RTD, read through domain; RdRp, RNA-dependent RNA polymerase. The sequenced PCR product from A and B did not have any significant matches to ApGlV2 or the NCBI database. PCR product C hit a pea aphid prestin-like transcript variant 5.
