# Supplementary material for: Aphis Glycines Virus 2, a Novel Insect Virus with a Unique Genome Structure
Source: Viruses. 2016 Nov 19;8(11):315. doi: 10.3390/v8110315 (PMC5127029; doi:10.3390/v8110315)
Supplement: Supplementary file 1 [file viruses-08-00315-s001.pdf]

Supplementary Materials: Aphis glycines virus 2, a Novel Insect Virus with a Unique Genome Structure

Sijun Liu, Diveena Vijayendran, Yuting Chen and Bryony C. Bonning

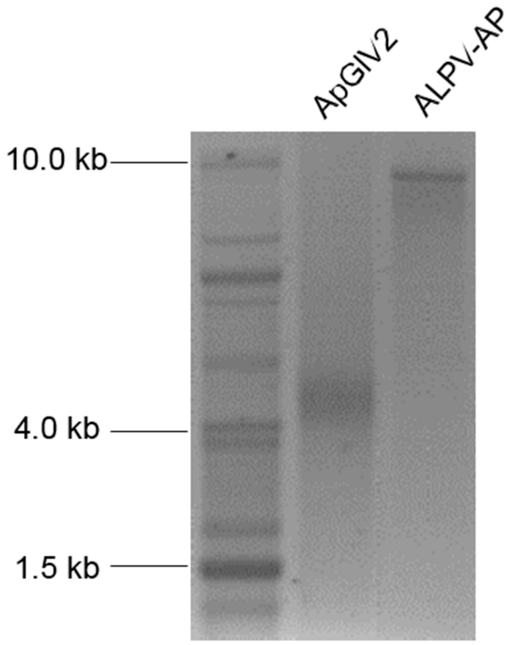

Figure S1. Gel electrophoresis (1% native agarose) of 2 μg RNA extracted from purified ApGlV2 and ALPV-AP virions. While the ALPV-AP RNA runs at the expected size of ~10 kb, the ApGlV2 RNA runs as a smear indicative of RNA degradation. Samples visualized with ethidium bromide staining.

Peptide 4 (sequencing coverage 3.69%)

```
MTRRTKKRAN TTKTMEKALV TAMERLTTKR RVPRKKGCAR VAEGEMVLRR EEMLVDVTLS
ANKTDSTGSV VLALANFPWL KTVAGSFERY KWKRLNIHWR AAGGFNKGGL IAVGMDWSNQ
LSSAYTRQTL TSASERQKVL SLTPHMSLPI SSTSINKTLG LPIKMLNSRN WYDAAKTDDE
GAVGAIRYSA KCDSDTVERF IGEIWVDYEV VLQGTRAQLP PPPPPPPPKH TLLYYSFTEM
RYVITRGQGA TNNVNSRNSS GISQRVVDND FTLSTDSFSD TVELLDGRTY RLIPTVQTET
EDDAPLDPDF EGLLTNVVSP PEQYSNTAHL KFYPLRDILK FRLTDFQLNL HLALEVEVNY
PTDMEALAIL WDFSRCLVSP QVRTRRESVK SNKALFHCVA AGLYRYVPAT RPYFKVSVEW
FNTGISSALA YTMKTQIYAF GLFGDSRTQE GFGQIAPSRA IKKRPVVKGL TQFHRSKAML
LGY
```

Peptide 5 (Sequencing coverage 11.98%)

```
MTRRTKKRAN TTKTMEKALV TAMERLTTKR RVPRKKGCAR VAEGEMVLRR EEMLVDVTLS
ANKTDSTGSV VLALANFPWL KTVAGSFERY KWKRLNIHWR AAGGFNKGGL IAVGMDWSNQ
LSSAYTRQTL TSASERQKVL SLTPHMSLPI SSTSINKTLG LPIKMLNSRN WYDAAKTDDE
GAVGAIRYSA KCDSDTVERF IGEIWVDYEV VLQGTRAQLP PPPPPPPPKH TLLYYSFTEM
RYVITRGQGA TNNVNSRNSS GISQRVVDND FTLSTDSFSD TVELLDGRTY RLIPTVQTET
EDDAPLDPDF EGLLTNVVSP PEQYSNTAHL KFYPLRDILK FRLTDFQLNL HLALEVEVNY
PTDMEALAIL WDFSRCLVSP QVRTRRESVK SNKALFHCVA AGLYRYVPAT RPYFKVSVEW
FNTGISSALA YTMKTQIYAF GLFGDSRTQE GFGQIAPSRA IKKRPVVKGL TQFHRSKAML
```

Peptide 6 (Sequencing coverage 17.05%)

```
MTRRTKKRAN TTKTMEKALV TAMERLTTKR RVPRKKGCAR VAEGEMVLRR EEMLVDVTLS
ANKTDSTGSV VLALANFPWL KTVAGSFERY KWKRLNIHWR AAGGFNKGGL IAVGMDWSNQ
LSSAYTRQTL TSASERQKVL SLTPHMSLPI SSTSINKTLG LPIKMLNSRN WYDAAKTDDE
GAVGAIRYSA KCDSDTVERF IGEIWVDYEV VLQGTRAQLP PPPPPPPPKH TLLYYSFTEM
RYVITRGQGA TNNVNSRNSS GISQRVVDND FTLSTDSFSD TVELLDGRTY RLIPTVQTET
EDDAPLDPDF EGLLTNVVSP PEQYSNTAHL KFYPLRDILK FRLTDFQLNL HLALEVEVNY
PTDMEALAIL WDFSRCLVSP QVRTRRESVK SNKALFHCVA AGLYRYVPAT RPYFKVSVEW
FNTGISSALA YTMKTQIYAF GLFGDSRTQE GFGQIAPSRA IKKRPVVKGL TQFHRSKAML
```

Peptide 7 (Sequencing coverage 20.28%)

```
MTRRTKKRAN TTKTMEKALV TAMERLTTKR RVPRKKGCAR VAEGEMVLRR EEMLVDVTLS
ANKTDSTGSV VLALANFPWL KTVAGSFERY KWKRLNIHWR AAGGFNKGGL IAVGMDWSNQ
LSSAYTRQTL TSASERQKVL SLTPHMSLPI SSTSINKTLG LPIKMLNSRN WYDAAKTDDE
GAVGAIRYSA KCDSDTVERF IGEIWVDYEV VLQGTRAQLP PPPPPPPPKH TLLYYSFTEM
RYVITRGQGA TNNVNSRNSS GISQRVVDND FTLSTDSFSD TVELLDGRTY RLIPTVQTET
EDDAPLDPDF EGLLTNVVSP PEQYSNTAHL KFYPLRDILK FRLTDFQLNL HLALEVEVNY
PTDMEALAIL WDFSRCLVSP QVRTRRESVK SNKALFHCVA AGLYRYVPAT RPYFKVSVEW
FNTGISSALA YTMKTQIYAF GLFGDSRTQE GFGQIAPSRA IKKRPVVKGL TQFHRSKAML
```

Figure S2. Peptide sequences derived from ApGlV2 structural proteins. Peptide numbers correspond to protein bands in Figure 3B; Colors represent sequences of individual peptide fragments. Proteins were identified from these peptide sequences with high confidence based on Mascot search A2 values.

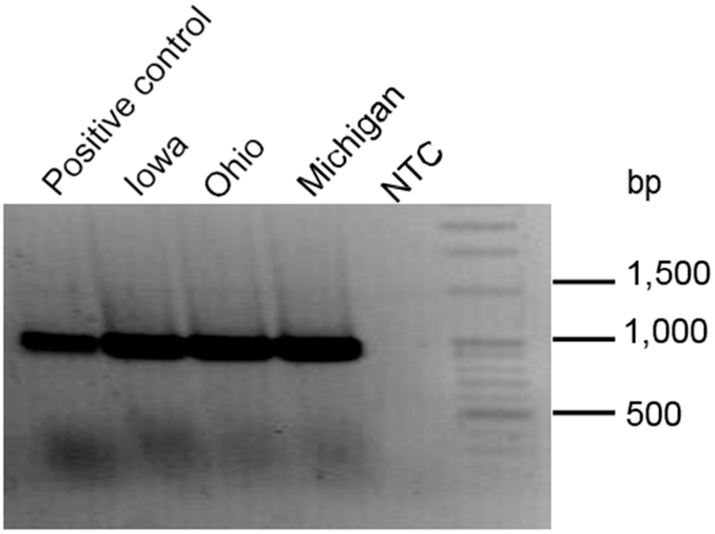

Figure S3. Detection of ApGlV2 RdRp sequence from field collected *A. glycines*. The 929 bp RT-PCR product was detected in field collected *A. glycines* from Iowa, Ohio and Michigan, USA. The positive control and *A. glycines* samples amplified the expected PCR products. No amplification was detected in the no template control sample (NTC).

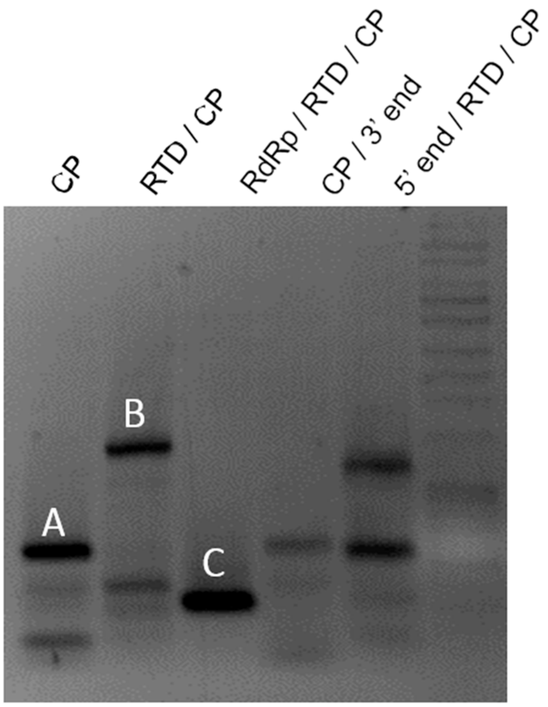

Figure S4. ApGlV2 sequences were not detected in the *A. glycines* genome. Primers spanning the entire ApGlV2 genome were used to test for the presence of ApGlV2 sequence in the *A. glycines* genome. A representative agarose gel with amplified PCR products is shown. Labels indicate region of ApGlV2 genome spanned by the PCR primers (Table 1). CP, capsid protein; RTD, read through domain; RdRp, RNA-dependent RNA polymerase. The sequenced PCR product from A and B did not have any significant matches to ApGlV2 or the NCBI database. PCR product C hit a pea aphid prestin-like transcript variant 5. Primers used: RdRp/CP (ApGlV2 R/C—F and ApGlV2 R/C—R to amplify 2976–3845 nt; 869 bp product): RTD/CP (ApGlV2 C/T—F and ApGlV2 C/T—R to amplify 3885–4445 nt; 561 bp product): RdRp/RTD/CP (ApGlV2 R/C—F and ApGlV2 C/T—R to amplify 2976–4445 nt; 1469 bp product): CP/3′-end (ApGlV2 C/T—F and APGLV2 3′—R to amplify 3885–4502 nt; 617 bp product): 5′ END/RTD/CP (ApGlV2 5′—F and ApGlV2 C/T—R to amplify 160–4445 nt; 4285 bp product).
